# Ischaemic colitis during pegylated interferon-alpha monotherapy for chronic hepatitis B: A case report

**DOI:** 10.1097/MD.0000000000033378

**Published:** 2022-04-07

**Authors:** Yu Liu, Hui Chen, Zhi-Ying Xu

**Affiliations:** a Intensive Care Unit, Hunan Provincial People’s Hospital, The First Affiliated Hospital of Hunan Normal University, Changsha, Hunan Province, China; b Department of Gastroenterology, Hunan Provincial People’s Hospital, The First Affiliated Hospital of Hunan Normal University, Changsha, Hunan Province, China; c Internal Medicine Ward, Hunan Provincial People’s Hospital, The First Affiliated Hospital of Hunan Normal University, Changsha, Hunan Province, China.

**Keywords:** adverse effects, hepatitis B, interferon, ischemic colitis, pegylated interferon-alpha

## Abstract

Pegylated interferon-alpha (PEG-IFN-α) is available for the treatment of hepatitis B virus infection, which is better than interferon-alpha (IFN-α) for the inhibition of hepatitis B virus replication. Ischemic colitis has been described from non-pegylated IFN-α, which occurs mainly in patients with hepatitis C virus infection. This is the first case of ischemic colitis during pegylated IFN-α monotherapy for chronic hepatitis B.

**Patient concerns:**

A 35-year-old Chinese man presented with complaints of acute lower abdominal pain and haematochezia, who was receiving PEG-IFN-α-2a monotherapy for chronic hepatitis B.

**Diagnoses:**

Colonoscopy revealed scattered ulcers and severe mucosal inflammation with edema in the left hemi colon and necrotizing changes in the descending portion. Biopsies revealed focal mucosal chronic inflammation and mucosal erosion. Therefore, the patient was diagnosed with ischemic colitis based on clinical and testing results.

**Interventions:**

PEG-IFN-α therapy was discontinued and switched to symptomatic management.

**Outcomes:**

The patient was discharged from the hospital after recovery. Follow-up colonoscopy revealed normal. The temporal association between the resolution of ischemic colitis and cessation of PEG-IFN-α treatment strongly favors the diagnosis of interferon-induced ischemic colitis.

**Lessons:**

Ischaemic colitis is a severe emergency complication of interferon therapy. Physicians should consider this complication in any patient taking PEG-IFN-α who develops abdominal discomfort and hematochezia.

## 1. Introduction

Drug-induced ischemic colitis (IC) is a serious condition. In its most severe form, complications of ischemic colitis include bowel infarction, necrosis, and rarely death.^[1]^ In published case reports, interferon-alpha has been suspected of inducing IC.^[2]^ Estimates of the incidence of IC associated with interferon-alpha (IFN-α) treatment vary from 0.3% to 0.7%, and it is most often seen in patients with chronic viral hepatitis C and multiple sclerosis.^[3]^ IFN-α is widely used to treat hepatitis B virus (HBV) infection, but no case of IC associated with interferon (IFN) treatment for chronic hepatitis B has ever been reported.^[1]^ Pegylated interferon-alpha (PEG-IFN-α) is more effective than IFN-α in the inhibition of HBV replication, and it can improve the serum liver fibrosis index.

This is the first case report of IC associated with PEG-IFN-α-2a monotherapy for chronic hepatitis B. Our aim is to increase awareness of this potential complication of antiviral treatment.

## 2. Case presentation

**Figure 1.**
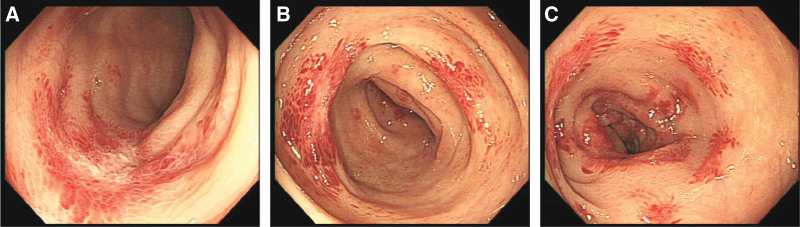
Colonoscopy 14 days before admission revealed lesions in the mucosa of rectum and sigmoid colon. (A) Sigmoid colon, (B) descending colon, and (C) transverse colon.

A 35-year-old Chinese man presented with complaints of acute lower abdominal pain and haematochezia. Fourteen days before admission, he reported having 10 to 20 bowel movements per day. They were diarrheal at first and became hemorrhagic later. A colonoscopy (Fig. 1) revealed lesions in the mucosa of rectum and sigmoid colon. He felt better after symptomatic treatment, so that he refused hospitalization. Eight hours before admission, the patient experienced recurrence of above symptoms, accompanied by acute lower abdominal pain when he was receiving PEG-IFN-α-2a therapy for chronic hepatitis B. He denied the presence of constipation, recent travel, and use of antibiotics or nonsteroidal anti-inflammatory drugs. He had no history of smoking, alcohol intake, hypertension, diabetes mellitus, dyslipidaemia, hypercoagulable state, atrial fibrillation, valvular heart disease, coronary artery disease or congestive heart failure.

On physical examination, his vital signs were within normal limits, with a heart rate of 78, respiratory rate of 20, and blood pressure of 130/81. Physical examination revealed left lower abdominal tenderness with no muscular guarding or rebound tenderness.

On admission, the patient’s hemoglobin was 127 g/L (normal values 113g/L to 151g/L). White blood cell count was 3.81 × 10^9^/L (normal values 3.69 × 10^9^/L to 9.16 × 10^9^/L) and platelet count was 65 × 10^9^/L (normal values 101 × 10^9^/L to 320 × 10^9^/L). Serum C-reactive protein, electrolyte levels, prothrombin time, partial thromboplastin time, liver enzymes, blood urea nitrogen and serum creatinine tests were within normal limits. Testing results of antinuclear antigen, T-SPOT, and HBV-DNA were negative. Stool cultures were negative.

**Figure 2.**
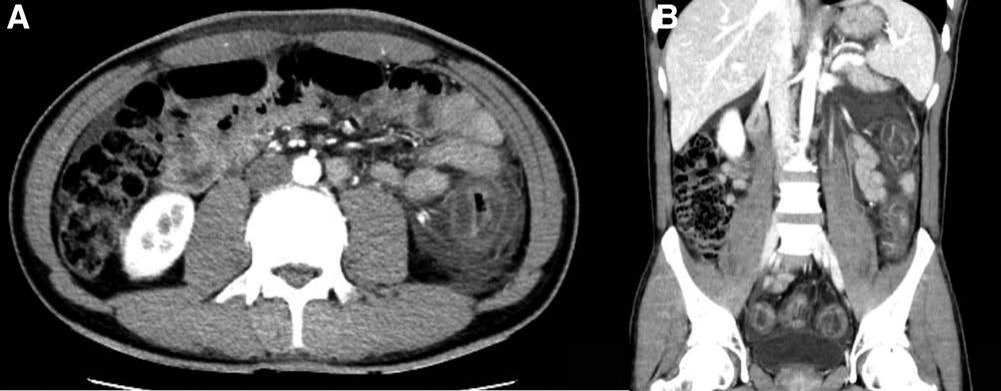
Oedema and thickening of the rectum and colon walls were observed on mesenteric computed tomography angiography at admission.

**Figure 3.**
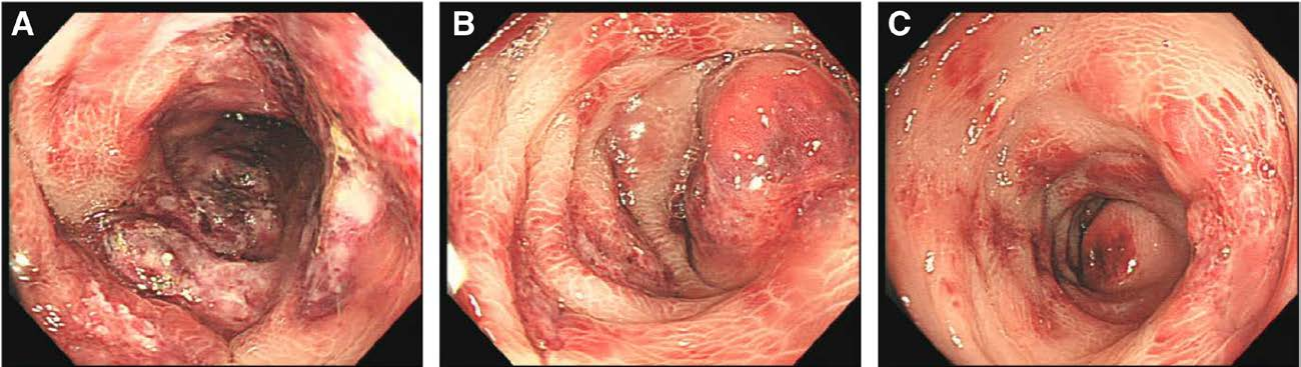
Colonoscopy at admission revealed scattered ulcers, severe mucosal inflammation with edema in the left hemi colon, and necrotizing changes in the descending colon. (A) Sigmoid colon, (B) descending colon, and (C) transverse colon.

**Figure 4.**
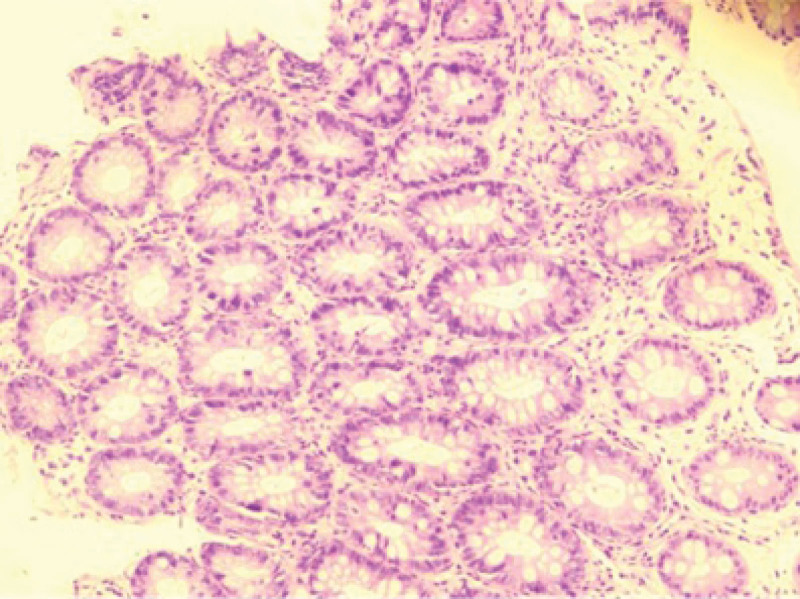
Biopsies of sigmoid colon at admission revealed focal mucosal chronic inflammation and mucosal erosion.

Oedema and thickening of the rectum and colon walls were observed on mesenteric computed tomography angiography, which indicated a high possibility of inflammatory reactions (Fig. 2). Colonoscopy revealed scattered ulcers, severe mucosal inflammation with edema in the left hemi colon and necrotizing changes in the descending colon (Fig. 3). Biopsies revealed focal mucosal chronic inflammation and mucosal erosion (Fig. 4). Therefore, the patient was diagnosed with IC based on clinical and testing results.

**Figure 5.**
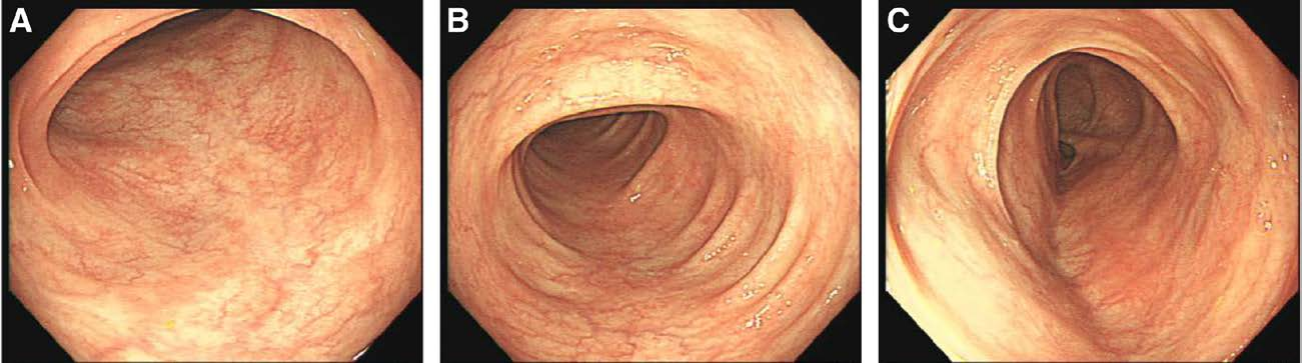
Complete resolution of IC was apparent on follow-up colonoscopy 2 mo after admission. (A) Sigmoid colon, (B) descending colon, and (C) transverse colon. IC = ischemic colitis.

After admission, PEG-IFN-α therapy was discontinued and switched to symptomatic management. Symptoms resolved after above treatment. An abdominal enhanced computed tomography revealed less intestinal wall edema and thickening than before. Complete resolution of IC was apparent on follow-up colonoscopy 2 months after admission (Fig. 5), suggesting a transient type of IC.

The patient did not have any predisposing conditions for IC, such as hypertension, diabetes mellitus, dyslipidemia, constipation or thrombophilia.^[1]^ Partial-thickness IC without multiorgan failure manifestations is usually self-limiting, however, the patient felt worse and worse after the first onset,^[4]^ which suggested that the cause had not been removed. The symptoms didn’t disappear until PEG-IFN-α therapy was stopped. The temporal association between the resolution of IC and cessation of PEG-IFN-α treatment strongly favored the diagnosis of IFN-induced IC.

## 3. Discussion

In published case reports, IFN-α has been suspected of inducing IC, mainly in patients with hepatitis C virus (HCV) infection, and HCV was also reported to increase the risk of IC^[5]^; IC associated with IFN treatment for chronic HBV infection has not been reported. To the best of our knowledge, this is the first case of IC associated with IFN treatment for chronic hepatitis B, and the first case attributable to PEG-IFN-α monotherapy.^[6]^

To our knowledge, the molecular mechanisms underlying PEG-IFN-α-associated ischemic colitis has not been established, while ischemic colitis has not been reported with other Peg proteins (e.g., pegvisomant, pegfilgrastim or pegademase), suggesting that the inert, polyethylene glycol moiety of PEG-IFN-α is unlikely to be the causative agent. The mechanisms of IFN-associated ischemic colitis is also unclear, but the following 3 possibilities exist. First, gastrointestinal vasculitis because of the immune-modulating effects of IFN may cause ischemic colitis. IFN increases immunity and subsequent deposition of immune complexes in the vascular wall, which may cause gastrointestinal vasculitis and ischemic colitis. Second, combination treatment with interleukin-2 and IFN may cause ischemic colitis, as reported by Sparano et al. Cytokine networks are activated by IFN, which may result in endothelial injury via superoxide radical generation. Thus, up-regulated thrombogenic effects activated by IFN-induced cytokines may be a candidate cause of ischemic colitis. Third, IFN is known to have a direct vasospastic effect, leading to a reduction in blood flow. In fact, Sasaki et al reported a significant decrease in regional cerebral blood flow during IFN therapy, suggesting that IFN-induced vasospasm may be responsible for the development of ischemic colitis.^[7–15]^ Clearly, additional study of the molecular mechanisms behind IFN-induced ischemia is warranted.

## 4. Conclusion

Ischemic colitis has been described from non-pegylated IFN-α, which occurs mainly in patients with HCV infection. This is the first case of ischemic colitis induced by PEG-IFN-α monotherapy in a patient with HBV infection. Physicians should consider this complication in any patient taking PEG-IFN-α who develops abdominal discomfort and hematochezia.

## Author contributions

**Supervision:** Zhi-Ying Xu.

**Writing – original draft:** Yu Liu.

**Writing – review & editing:** Hui Chen.
